# Pathological characteristics of traditional Chinese medicine *Shi Zheng* and related therapeutic formula, effective compounds: a general review

**DOI:** 10.1186/s13020-025-01196-w

**Published:** 2025-12-08

**Authors:** Ying Wang, Le Yang, Hui Sun, Ye Sun, Guangli Yan, Ying Han, Xijun Wang

**Affiliations:** 1 State Key Laboratory of Dampness Syndrome, The Second Affiliated Hospital Guangzhou University of Chinese Medicine, Dade Road 111, Guangzhou, China; 2 State Key Laboratory of Integration and Innovation of Classic Formula and Modern Chinese Medicine, Metabolomics Laboratory, Department of Pharmaceutical Analysis, National Chinmedomics Research Center, National TCM Key Laboratory of Serum Pharmacochemistry, Heilongjiang University of Chinese Medicine, Heping Road 24, Harbin, 150040 China

**Keywords:** *Shi Zheng* (Dampness syndrome), Chinese medicine formulas, Effective compounds, Rheumatoid arthritis, Nonalcoholic fatty liver disease, Gouty arthritis

## Abstract

*Shi Zheng* (Dampness syndrome) is a prevalent condition in traditional Chinese medicine (TCM) syndrome caused by the humid environment (external dampness) or metabolic imbalance (internal dampness) and characterized by sense of heaviness in the body and numbness in the limbs. Most *Shi Zheng* patients suffer from metabolic disorders and inflammation, and they were diagnosed as the diseases such as rheumatoid arthritis, gouty arthritis, nonalcoholic fatty liver disease or type 2 diabetes mellitus by modern medicine, and they are prone to complications or recurrent episodes despite long-term medication. Chinese medicine formulas (CMFs) and their effective compounds have shown promising results in treating these diseases, with high cure rates and a low incidence of adverse events. However, modern science has yet to establish a clear understanding of the underlying mechanisms between *Shi Zheng*, related diseases, and CMFs, probably because of the extremely abstract concept of TCM syndrome. Therefore, this review aims to provide an overview of the characteristics of *Shi Zheng* and the effects of CMFs and active compounds in TCMs on typical diseases associated with *Shi Zheng* to clarify the concrete connection between TCM symptoms and modern diseases, thereby to bridge the gap between TCM syndrome concepts and modern medicine.

## Introduction

*Shi Zheng* (Dampness syndrome) is a traditional Chinese medicine (TCM) syndrome induced by damp factors and represents a unique pathophysiological reaction state in TCM [1, 2]. *Shi Zheng* can occur in various parts of the human body and can combine with other TCM conditions to induce pathological changes [3, 4]. Many modern diseases, such as arthritis, IgA nephropathy, chronic hepatitis B, nonalcoholic fatty liver disease (NAFLD), and chronic gastritis, are closely related to *Shi Zheng* in the diagnosis and classification of TCM [5–7]. These diseases are notoriously difficult to cure as chronic diseases and even persistently induce a micro-inflammatory state in the body [8–14]. Thus, clarifying the etiology and therapeutic strategies of *Shi Zheng* is of paramount importance. In general, the Chinese medicine formulas (CMFs) with diuretic, sweat-inducing, and dampness-draining effects have been shown to be effective in treating diseases caused by external and internal dampness, such as arthralgia and swelling, and reducing the risk of fracture in rheumatoid arthritis (RA) patients [15–17]. However, due to the complex and individualized nature of CMFs and the lack of clear pharmacodynamic mechanisms, TCM is often used as a supplementary alternative therapy in the treatment of these diseases. Patients typically opt for chemical drugs or surgical interventions [18].

**Fig. 1:**
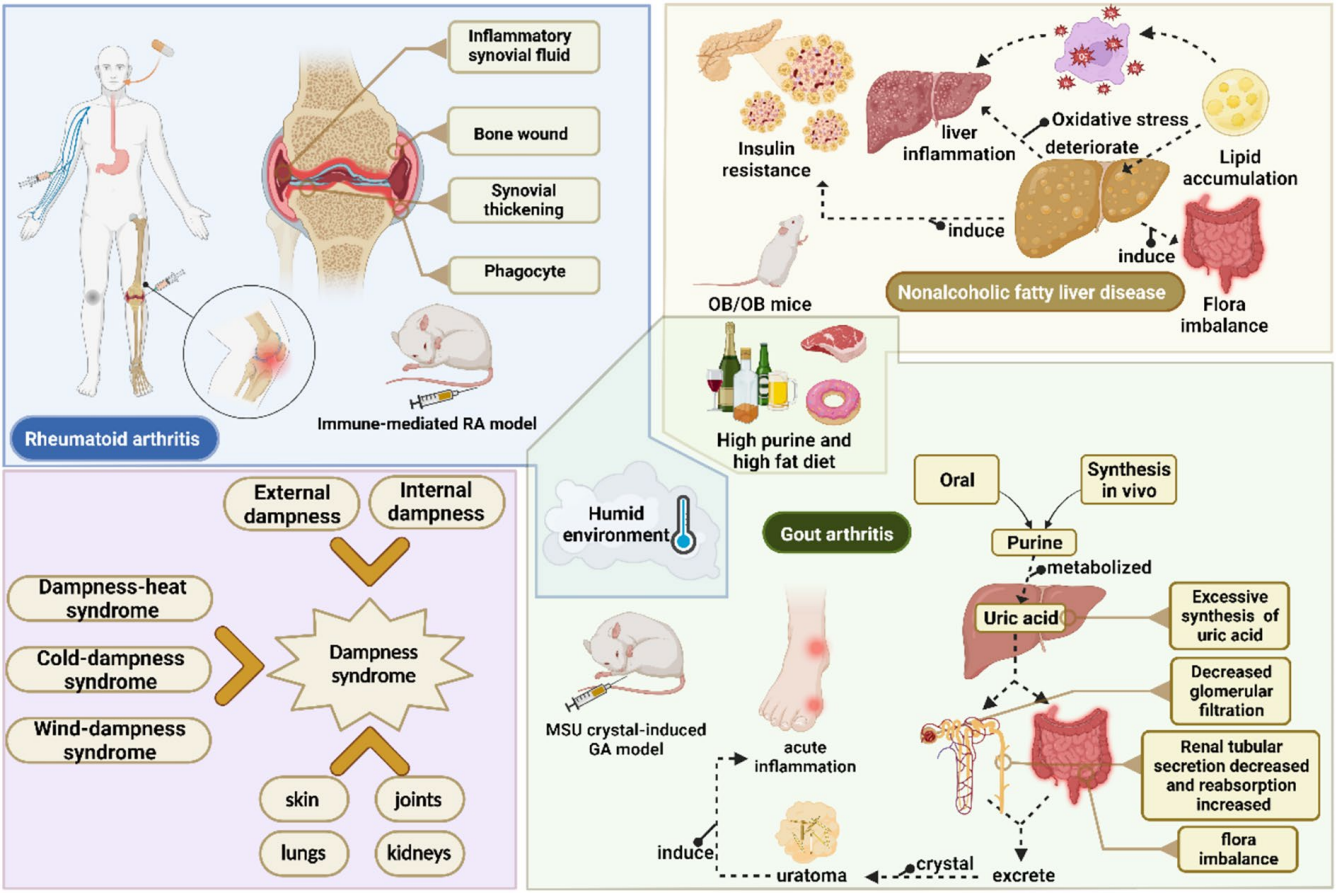
The relationship between *Shi Zheng* and its crucially related diseases

Currently, extensive research has illuminated the diversity and characteristics of the essential pathogenesis of Shi Zheng, which can manifest in various typical diseases. The pathogenesis of TCM arthralgia syndrome encompasses external dampness and cold weather, and patients with this syndrome exhibit symptoms akin to those of RA patients with *Shi Zheng* [19–21]. In modern medicine, RA is classified as an autoimmune rheumatic disease with an age-standardized incidence rate in China of approximately 13.7% [22]. It is characterized by obvious metabolic disorders and a decreased survival rate [23–25]. Patients with mild or moderate RA typically require early treatment and long-term medication to alleviate discomfort. However, discontinuing medication can potentially exacerbate the condition, and in severe cases, joint replacement surgery emerges as an effective clinical treatment option [26–29]. Notably, nonalcoholic fatty liver disease (NAFLD), recognized as a typical "Ganpi"disorder in TCM, includes hepatic pain and swelling caused by hepatic fat accumulation due to spleen deficiency and internal dampness, usually resulting from unhealthy lifestyle habits [30]. Metabolic imbalances, intestinal microbial imbalances, and abnormal immune responses in NAFLD patients contribute to liver lipid accumulation and inflammation, ultimately leading to the development of cirrhosis and other severe complications, which can result from hereditary or other factors [31]. Lifestyle adjustments represent the primary means of managing early-stage NAFLD, and in later stages, small molecular regulators such as vitamin E and pioglitazone are employed to treat inflammation and fibrosis [32]. However, the limited availability of early targeted treatment strategies poses significant challenges for NAFLD treatment. Moreover, gouty arthritis (GA) classified as "lijiefeng" or "baihufeng" within TCM, is characterized by deficiency, internal dampness, and external dampness [33, 34]. Clinical research has revealed that individuals with obesity and metabolic disorders are more susceptible to GA, which is associated with high blood uric acid (UA) levels and inflammatory reactions in multiple joints, which result from abnormal purine metabolism. The accumulation of monosodium urate (MSU) can lead to bone erosion and even renal injury as the disease progresses [35]. Anti-inflammatory agents (e.g., NSAIDs, colchicine, glucocorticoids), uric acid excretion-promoting agents (e.g., allopurinol), and uric acid production-inhibiting agents (e.g., febuxostat and benzbromarone) have demonstrated significant efficacy in treating GA, albeit with potential adverse reactions [36]. Clinically, TCMs have demonstrated positive effects on the pathology of these diseases and cause fewer adverse events than Western combined therapy [37–47]. Consequently, TCMs represent a promising therapeutic strategy for treating diseases caused by *Shi Zheng* (Fig. 1).

*Shi Zheng*, a quintessential TCM syndrome, is associated with various pathological changes. The efficacy and safety of CMFs and effective compounds from TCMs have been substantiated in the treatment of various diseases caused by *Shi Zheng*. However, the critical role of TCM syndromes in disease treatment should be clearly defined to further elucidate the rationality of TCM theory. Thus, focusing on the characteristics of *Shi Zheng* and the application of TCMs in clinical practice, we selected RA, NAFLD, and GA as exemplars and systematically reviewed animal models that replicate key characteristics of TCM diagnosis, as well as the therapeutic effects and mechanisms of TCMs in these models. This study aims to explicitly demonstrate the significant impact of TCMs on the treatment of diseases caused by *Shi Zheng* and to provide evidence-based recommendations for the rational treatment of such diseases and the development of novel therapeutic strategies (Fig. 1).

## Cognition of *Shi Zheng* in TCM

### Pathogenesis mechanism

*Shi Zheng* syndrome is caused by external or internal dampness, each of which has distinct pathogenic mechanisms (Fig. 1) [48]. Moreover, *Shi Zheng* has been found to trigger inflammatory reactions of varying degrees and modulate immune function. Internal dampness rapidly impairs T-cell immune recognition without significantly elevating inflammatory markers; in contrast, external dampness tends to upregulate inflammatory factors, compared to internal dampness, external dampness is more likely to induce inflammation [49, 50]. For instance, atopic dermatitis, an inflammatory skin condition caused by a humid environment (external dampness), can be effectively managed by the dampness-eliminating effect of Huoxiang Zhengqi [51]. Erchen Decoction has been shown to ameliorate lipid metabolism and oxidative stress-related pathways, while Huo-Tan-Chu-Shi Decoction has demonstrated efficacy in alleviating myocardial ischemia, hypertrophy, and fibrosis associated with coronary heart disease (CHD) combined with phlegm-dampness syndrome [18, 52].

### Involvement Zang-Fu

Multiple Zang-Fu can be impacted by *Shi Zheng* when invaded by a damp pathogen (Fig. 1). TCM theory suggests that spleen is a vital organ for transporting water and fluid metabolism, and Spleen Qi deficiency can result in the accumulation of water-dampness, dampness-obstructing spleen-stomach syndrome, and impairs the function of the intestinal barrier, which can be improved by Huoxiang Zhengqi oral liquid [51, 53, 54]. Additionally, *Shi Zheng* can easily affect joints, skin, lungs, and kidneys, leading to conditions such as arthritis, urticaria, COVID-19, and diabetic nephropathy. According to TCM clinical diagnosis, Qingluo San, oral Guben Xiaozhen prescription, Sanren decoction, Taohong Siwu decoction, and Wuling Powder have been found to be effective at treating these syndromes [4, 55–58].

### Comprehensive syndrome

The *Shi Zheng* patients often experience invasion by wind, cold, or heat pathogens, leading to the development of dampness-heat syndrome, cold-dampness syndrome, or wind-dampness syndrome with  high phthogenicity (Fig. 1). Some patients with hepatic diseases, jaundice, inflammatory diseases, or cancer have been diagnosed with dampness-heat syndrome in TCM clinical practice [59–63]. For example, dampness-heat tends to accumulate in the liver and gallbladder to  induce liver-depression and hypochondriac pain and identified as a category of modern disease hepatitis. Hepatitis patients were diagnosed as multiple types of dampness-heat syndrome by TCM. Modern research has confirmed that dampness-heat syndrome possibly induces serious liver damage,  immune response differences, disruptions in the biosynthesis of secondary metabolites, microbial metabolism in various environments, the carbon fixation pathway in prokaryotes, protein digestion and absorption, and carbohydrate digestion and absorption, and differential expression of key miRNA (such as hsa-miR-483-3p and hsa-miR-223-3p) [6, 7, 64–66]. Furthermore, patients with this syndrome exhibit similar features in their intestinal microflora, as evidenced by the correlation between the syndrome and communities of *Agathobacter, Dorea, Lachnospiraceae_NC2004_group*, *Subdoligranulum*, and *unclassified_c_Clostridia*, as well as *Ruminococcus_gnavus_group* [67]. Yinchenhao decoction and Rong-Yang-Jyh-Gan-Tang have proven to be effective treatments for dampness-heat hepatitis patients [68, 69]. Patients with dampness-heat hepatitis are prone to jaundice, which can be effectively treated with Zhizi Baipi decoction, Yinchenhao decoction, and the Jigucao capsule [70–72]. In addition, dampness-heat easily invades joints, kidneys, lungs or bladder, leading to inflammatory diseases such as GA, chronic nephritis, pneumonia and simple lower urinary tract infection, which can be treated with TCM formulae such as Qinpi Tongfeng Formula, compound Qingbi granules, Jianpi Qinghua Prescription, Xiang Qin Kang Gan Granules, and Sanjin Tablets, respectively [61, 73–77].

Cold-dampness syndrome is caused by Yang deficiency and a humid environment and can result in diseases such as epidemics, diarrhea, primary dysmenorrhea, chronic pelvic inflammatory disease, chronic urticaria, and arthritis. Cold-dampness-induced infectious pneumonia, such as COVID-19, is classified as an epidemic disease. Among COVID-19 patients, 8.50% exhibited cold-dampness accumulation in lung syndrome patients, which was successfully treated with dampness-eliminating CMFs such as Huopu Xialing Decoction and Guizhi Decoction. An analysis on application law of dampness-removing traditional Chinese medicines in treatment of coronavirus disease 2019 [78–81]. Additionally, cold-dampness entering the uterus directly causes abdominal pain in females or abdominal pain during menstruation, particularly primary dysmenorrhea and pelvic inflammation. 68.15% of primary dysmenorrhea patients suffer from cold and dampness stagnation. The Danggui Sini Decoction has shown efficacy in relieving long-term cold-induced pain [82–84]. CMFs that eliminate cold and dispel dampness, such as Shaofu Zhuyu Decoction and Wenjing Decoction, have been found to be effective at alleviating symptoms such as abdominal distension, increased leucorrhea, fatigue, and inflammation in chronic pelvic inflammatory patients with cold-dampness syndrome, which is classified as below-band disease and abdominal pain in TCM [85, 86]. Chronic urticaria, classified as cold-dampness rubella in TCM, can be treated by reducing itching, wheal, and attack frequency through the use of the Mahuang Fuzi Xixin Decoction combined with Wuling Powder [87]. Patients with cold-dampness arthralgia osteoarthritis, such as RA, ankylosing spondylitis, and knee osteoarthritis, are relieved by warming Yang, tonifying the kidney, and removing the arthralgia effects of the Duhuo Jisheng decoction [5, 88, 89].

Wind-dampness syndrome is closely associated with renal diseases. The TCM classification of IgA nephropathy patient is mainly wind-dampness syndrome, characterized by tiredness and pain in the waist; soreness of the head, body, muscles, and joints; eczema; and aversion to wind. These patients have shown improvements in CMFs that dispel wind and dampness, such as Radix *Stephania Tetrandra*, *Cynanchum paniculatum* Radix, and *Sinomenii Caulis*, which have a high remission rate for urinary protein [90, 91]. Moreover, wind-dampness syndrome has been found to affect metabolite levels and disease severity. For instance, compared with other patients, patients with wind-dampness syndrome glomerulopathy exhibit significantly greater IgGCR, TCR, ACR, and α1CR. In addition, wind-dampness syndrome lupus nephritis patients are more likely to experience fever, serositis, edema, hypertension, and a high lupus erythematosus disease activity index [92, 93].

## Rheumatoid arthritis and *Shi Zheng*

### Rheumatoid arthritis

Rheumatoid arthritis (RA) is a chronic autoimmune disease with an inflammatory state. In the early stages of RA, metatarsal bursitis occurs, and as the disease progresses, angiogenesis, synovial hyperplasia, inflammation of the matrix, and destruction of cartilage and bone tissue are observed (Fig. 1) [94, 95]. Besides, the RA disease activity in patients is potentially associated with the increased risk of renal dysfunction including alterations in glomerular filtration rate [96]. Modern investigations demonstrated that NF-κB receptor activator, anti-citrulline protein antibodies (ACPAs) and IL-34 effectively promote osteoclast formation and induce bone erosion [97]. The expression of serum proteins in RA patients can distinguish between positive and negative ACPA patients, as well as ACPA-mediated osteoclast activation and nociceptive chemokine CXCL1/7 [98–100]. Recent research has suggested that IL-34 may be a novel biomarker for predicting bone erosion in RA; however, there is still limited research on predictors and diagnostic criteria for the early onset of RA [101]. Glucocorticoids, immunosuppressants and non-steroidal anti-inflammatory drugs are commonly employed in the clinical treatment of RA for their anti-inflammatory, immunosuppressive, and analgesic properties, forming the cornerstone of routine treatment regimens [102]. However, in the course of treatment, combined medication is usually needed to relieve the symptoms of RA, but including severe hepatorenal toxicity and cardiovascular damage [103]. The integrating traditional Chinese medicine (TCM) prescriptions with conventional therapeutic drugs can significantly enhance efficacy and safety. For instance, the combination of GuiZhi-ShaoYao-ZhiMu decoction, a well-known TCM formula, with methotrexate can reduce the joint swelling and tenderness, the duration of morning stiffness, the level of C-reactive protein, rheumatoid factor and erythrocyte sedimentation rate in patients with RA. This highlights the potential advantages of incorporating TCMs into the treatment of RA [104–112].

The high cure rate of TCMs for RA is strongly related to its dialectical treatment. TCM considers RA to fall under the category of arthralgia syndrome, which is characterized by pain and numbness in the limbs, joints, skin, bones, and muscles caused by externally-contracted wind, cold, dampness, or heat pathogens [3]. Furthermore, 67.6% of RA patients were diagnosed with *Shi Zheng*, which included cold-dampness obstruction, wind-dampness obstruction, and dampness-heat obstruction [113, 114]. The diversity of TCM syndromes in RA patients leads to differences in metabolites and blood cells. Metabolites, such as C17-sphinganine and leucyl-alanine, demonstrate high diagnostic efficacy in RA patients presenting with heat-dampness syndrome and kidney-liver deficiency syndrome [115]. The erythrocyte sedimentation rate, C-reactive protein level, white blood cell count, platelet count, and globulin level in the serum, as well as the IL-17 level in synovial fluid, are greater in dampness-heat obstruction RA patients than in cold-dampness obstruction RA patients [116, 117]. Classic TCMs effectively improve clinical symptoms in dampness pattern RA patients with minimal adverse reactions, providing evidence for the efficacy and safety of TCM for treating RA [118].

**Fig. 2:**
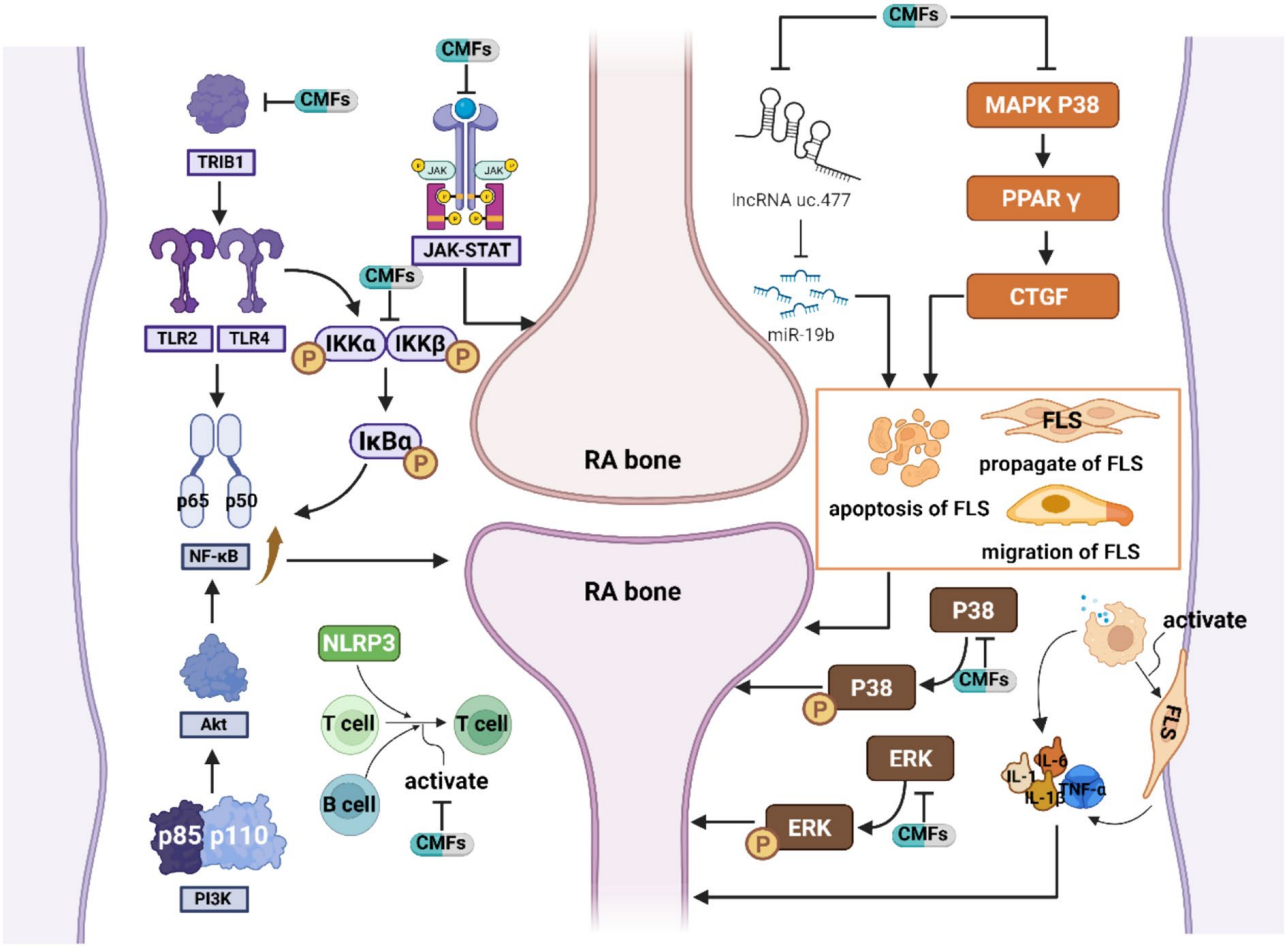
The mechanism of CMFs and their active substances for inhibition of RA inflammation

These include BiZhong Xiao decoction, Ermiao powder, and Guizhi Baihu decoction for dampness-heat obstruction, as well as classic prescriptions such as Duhuo Jisheng decoction, Wutou decoction, Qianghuo Shengshi decoction, and Juanbi decoction. Clinical experience prescriptions such as Huayu Qiangshen Tongbi decoction and Wenjinghuoluo prescription, as well as TCM patent drugs such as Zhuifeng Tougu capsule, Biqi capsule, and Fengshigutong capsule, have also shown efficacy in treating RA with wind-cold-dampness obstruction [89, 119–130]. These CMFs commonly employ TCMs with dispelling wind and dampness function, generally including active ingredients  for RA such as triterpenoid saponins, alkaloids, phenolic acids, glycosides,  cyclohexene ether terpenoids, coumarins, flavonoids, saponins, and tannins [131, 132]. Such CMFs and active ingredients can effectively improve inflammation caused by RA, inhibit pannus formation, reduce the density of small, medium and large blood vessels in inflammatory tissues, and improve synovial hyperplasia and bone destruction (Fig. 2) [129, 130, 133–135].

### Inflammation

Inflammatory responses in RA are typically induced by the immunoreaction mediated by inflammatory cytokines such as IL-1, IL-6, and TNF-α in the initial stage of the disease. Thus, the level of inflammatory cytokines plays a crucial role in the development and treatment of RA. The secretion of inflammatory cytokines is regulated by pathways including the nuclear factor kappa-B (NF-κB) pathway, the Janus kinase/signal transducers and activators of transcription (JAK/STAT) pathway, oxidative stress, and the mitogen-activated protein kinase (MAPK) pathway [136]. Fibroblast-like synoviocytes (FLSs) in tissues act as key effector cells of RA and secrete inflammatory mediators upon activation. Additionally, macrophages, specifically the classically activated M1 (proinflammatory) phenotype, can produce inflammatory factors, activate FLSs, and promote bone destruction in RA. Conversely, the alternatively activated M2 (anti-inflammatory) phenotype of macrophages can facilitate synovial tissue repair in RA.

The NF-κB pathway plays a crucial role in various diseases, such as immune disorders, inflammation, and tumors. The Rel-A/NF-κB1 (p65/p50) heterodimer, one of the classical subtypes of NF-κB, consists of Rel-A (p65) and NF-κB1 (p50). It has been found to be significantly upregulated in the early stage of RA in patients who are receiving inadequate treatment [137]. This upregulation indicates the proinflammatory effect of NF-κB and highlights its potential as a target for anti-inflammatory drugs in RA treatment. Certain TCMs or their active components have shown potential for alleviating inflammation in RA by modulating the level of NF-κB, such as Jinteng Qingbi granules [138], TRIB1/TLR2/4 and PI3K/Akt are the main upstream regulatory factors [139]. For instance, Zhuifeng tougu capsules relieved arthritis symptoms in wind-cold-dampness-induced RA rats by modulating the TLR2/4-NF-κB pathway [128]. Icariin [140] and magnoflorine [141], Sappanone A [142], Guizhi Shaoyao Zhimu Decoction [143] have also been found to affect RA-related inflammation through the TRIB1/TLR2/NF-κB pathway and the PI3K/Akt/NF-κB pathway, respectively. Additionally, NF-κB is activated by phosphorylation of IKK and IκB to promote the expression of inflammatory factors (such as TNF-α) and their synthetases and genes (including COX-2 and iNOS), and the polarization of M1 macrophages, thereby exacerbating to the exacerbation of inflammation [144]. Animal experiments have shown the beneficial effects of certain CMFs, such as Xiaoyao-Qingluoyin [145], Fangji Huangqi Decoction [146] and Modified Xianfang Huoming Yin [147] as well as specific effective parts of TCMs, such as polar extracts of Rhododendron molle G. Don leaves [148] and notoginsenoside R1 [149]. These interventions inhibit the production and secretion of inflammatory factors to suppress the activation of NF-κB. Furthermore, NF-κB promotes the activation of NLR family pyrin domain-containing 3 (NLRP3) inflammasomes, resulting in the maturation and secretion of proinflammatory cytokines and the differentiation and activation of CD4 + T cells [144]. Jingfang Granules [150] and the active components of TCMs, including mangiferin, cinnamic acid [151], wedelolactone [152], and lonicerin [153], have been shown to inhibit inflammation by suppressing the activation of NLRP3 through the inhibition of NF-κB activation, and the release of IL-1β and IL-18.

The JAK/STAT pathway is integral to the development of RA. JAK with inhibition effect on the signal transduction of IL-6 as a non-receptor tyrosine kinase, including JAK1, JAK2, JAK3, and Tyk2 types [154]. Once activated, STATs (including STAT1, STAT2, STAT3, STAT4, STAT5A, STAT5B, and STAT6) bind to phosphorylated receptors, undergo phosphorylation by JAK, translocate into the nucleus, and bind with deoxynucleotides to activate the transcription of target genes. STAT3, in particular, plays a key role in RA angiogenesis by promoting the expression of metalloproteinases (MMP-2 and MMP-9) [136]. Wang-Bi tablet has shown significant efficacy in treating RA due to its activation of STAT3. Additionally, modified Xianfang Huoming Yin [146] and aconitum alkaloids from *Aconiti Radix Cocta* [155] alleviate RA-related inflammation, although the specific signaling factors involved remain unclear.

Among the MAPK family members, MAPK p38, extracellular signal-regulated kinase (ERK), and c-Jun N-terminal kinase (c-JNK) have been shown to influence RA development. MAPK p38 inhibits peroxisome proliferator-activated receptor γ (PPAR γ) activation and connective tissue growth factor (CTGF) expression. Shentong Zhuyu Decoction regulates the proliferation, migration, invasion, and apoptosis of RA-FLSs through the MAPK p38/PPAR*γ*/CTGF pathway [156]. Sinomenine [157], imperatorin [158], rutin, asperosaponin VI [159], Fuhu Lijie Tang [160], and berberine [161] inhibit key factors in the MAPK pathway, such as p-ERK, p-P38, p-STAT-1/3, p-PI3K, p-Akt, p-JNK, p-IκB, p-NF-κB, and β-catenin, thereby improving inflammation in RA.

Furthermore, inflammation in RA is closely associated with imbalances in immune cell subsets [162] and the expression of specific genes [163]. Xinfeng capsule [164] and Huayu Qiangshen Tongbi Decoction [165] alleviate RA inflammation by modulating the expression of apoptosis-related genes (lncRNA MK5-AS1) and the inflammation-related genes (lncRNA uc.477 and miR-19b) individually. In particular, the binding of miR-19b to TNF receptor superfamily 12 A (TNFRSF12A) leads to decreased expression of IL-6 and IL-1β induced by TNF-α.

### Angiogenesis

The proliferation of immature blood vessels in the synovium driven by vascular endothelial growth factor (VEGF) could be a characteristic of the chronic phase of inflammation in RA [166, 167]. VEGF is secreted from synovial tissue fibroblasts and stimulated by angiogenic factors (TNF-α, IL-1β, IL-17, TGF-β, PDGF, PIGF, and MMP), which  regulate the migration, invasion, adhesion, tube assembly, and remodeling of endothelial cells [168]. The proliferation, migration, and sprouting of endothelial cells are tightly linked to the signaling cascades between VEGF and VEGF receptor 2 (VEGFR2). ANG1 stabilizes newly formed blood vessels, which are further enhanced by the combination of pericytes and the newly formed basement membrane, promoting blood flow [169]. Matrine, and total saponins of *Panax japonicus* C.A. Meyer [170]*,* Clematichinenoside AR [171], inhibit angiogenesis through the hypoxia-inducible factor (HIF)-VEGF-ANG axis [172].

The PI3K/Akt and MAPK pathways are critical for cell proliferation, migration, invasion, and the production of proinflammatory cytokines [168]. Wutou decoction significantly inhibited the expression of HIF-1α and regulated the PI3K-AKT-mTOR-HIF-1α pathway to improve RA angiogenesis [169]. Several CMFs and their active ingredients, such as Kunxian Capsule [168] and Shikonin [173], can regulate the gene expression of PI3K and Akt. Additionally, inhibition of the LOX/Ras/Raf-1 pathway may be the mechanism of action of CMFs in RA. Yu-Xue-Bi tablets, a proprietary Chinese medicine, have been shown to ameliorate joint injury and synovial angiogenesis in CIA rats and inhibit LOX/Ras/Raf-1 signal transduction [167].

### Synovial hyperplasia and destruction of cartilage and bone tissue

Synovial hyperplasia and destruction of cartilage and bone tissue are typical pathological manifestations of RA and are characterized by the infiltration of synovial cells by inflammatory cells and their gradual invasion of bone tissue [174]. Serum metabolites and cytokines are key factors for evaluating bone and synovial lesions [175]. Currently, based on big data analysis and core plasma-metabolite profiles, a model has been developed to predict imaging progression, which provides a diversified new tool for early prediction of bone destruction in RA patients [176]. The Shexiang-Wulong pill, mentioned in Moschus Yuan, has been found to inhibit the secretion of proinflammatory cytokines such as TNF-α, IL-6, and IFN-γ, thereby alleviating synovial hyperplasia [177]. Furthermore, Baihu Jia Guizhi decoction inhibited the succinate/SUCNR1/IL-1β pathway, which is related to inflammation, and improved synovial hyperplasia in RA rats. SUCNR1, an important factor in immunocyte inflammation and antigen presentation enhancement by dendritic cells (DCs) induced by succinic acid, is involved [178]. Synovial hyperplasia is caused by the abnormal migration and proliferation of synovial cells. SLC3A2, a type II transmembrane protein, acts as an integrin accessory receptor, mediating integrin β3-dependent cell migration and promoting the interaction between integrins and the focal adhesion kinase/Src. Yishen Tongbi decoction inhibits the high expression of SLC3A2 and integrin β3 in the cell membrane and cytoplasm of synovial proliferative cells in the ankle joint of RA mice, thus alleviating synovial hyperplasia caused by RA [179].

**Fig. 3:**
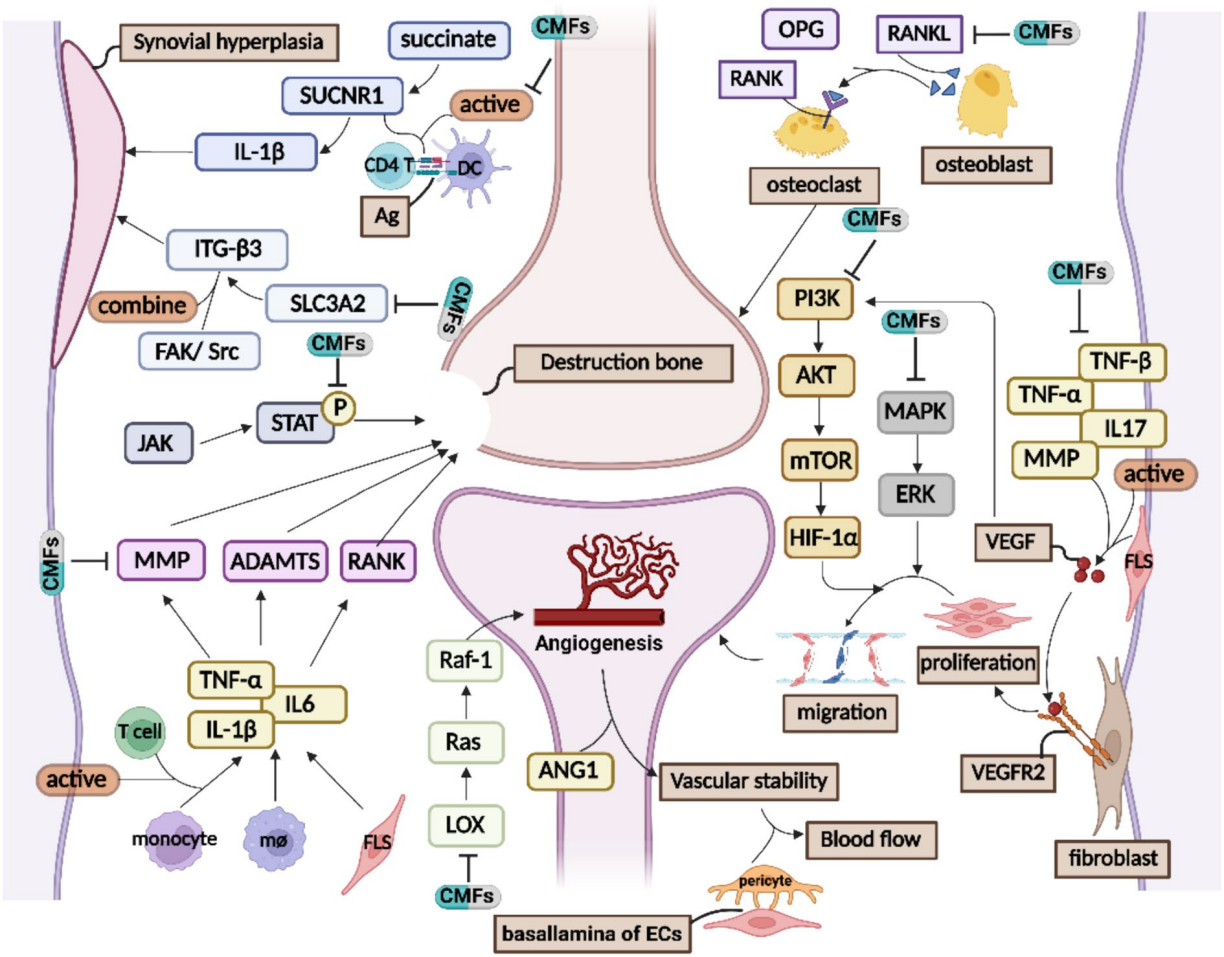
The mechanism of CMFs and their active substances for inhibition of RA angiogenesis and destruction of bone tissue

Bone destruction in RA primarily occurs due to a deficiency in osteoblast-mediated bone formation or an excess of osteoclast-mediated bone resorption, which is induced by inflammation and ultimately leads to bone loss. The evaluation of bone formation, bone resorption, and bone destruction in RA can be performed using osteoprotegerin (OPG) and receptor activator of nuclear factor-κB ligand (RANKL) [180]. The Wang-Bi capsule inhibits bone destruction by reducing the number of osteoclasts and adjusting the balance between OPG and RANKL [181]. Furthermore, proinflammatory cytokines play a critical role in bone destruction. They interact with T cells, enhancing their response to pathogenic antigens and activating monocytes, macrophages, and synoviocytes to produce more proinflammatory cytokines. These cytokines induce the production of matrix metalloproteinases (MMPs) and a disconnect and metalloproteinase with thrombospondin-like motifs (ADAMTSs), causing damage to connective tissue and joints. Binding to receptors triggers the production of receptor activator of NF-κB (RANK), which mediates osteoclast differentiation and further contributes to joint absorption and destruction [182]. Fufang Shatai Heji exerts a significant effect on bone destruction in RA by regulating MMP-13 and MMP-9 to prevent collagen degradation and downregulating the expression of ADAMTS-4 and ADAMTS-5 in chondrocytes to inhibit cartilage degradation [183]. Moreover, Wang-Bi Tablet [184], Wutou Decoction [185], Baixianfeng Decoction [186] and artemisinic acid [187] can refine bone destruction by modulating the expression of NF-κB. Additionally, the JAK-STAT pathway mediates the effect of the RANKL/RANK/OPG axis on bone destruction [188]. Simiao Pill inhibits joint inflammation to improve joint bone destruction by regulating the JAK2-STAT3 pathway (Fig. 3) [189].

### Animal model

Immune-mediated type RA models induced by Freund's adjuvant (FA) and type II collagen, spontaneous RA models of transgenic animals, and syndrome-combined disease RA models are commonly used animal model preparation methods in RA research (Table 1). These models are used to induce RA-related disease characteristics, such as joint swelling, pain, and deformity [190]. In the study of CMFs, it is important to assess the improvement in TCM symptoms as an indication of their effectiveness. Ge et al. developed a new RA model with TCM symptoms by stimulating female rats with environmental factors such as wind, humidity, heat, and cold. This model resulted in arthritis symptoms, increased inflammatory factor levels, synovial hyperplasia, and bone erosion in rats [191]. However, the simple TCM symptom model does not fully replicate the immune response process in the early stage of RA. To address this limitation, immune-inducing drugs can be used to induce an immune response in animals before the influence of the natural environment is simulated using an artificial climate box. This approach allows for the preparation of a syndrome-combined disease RA model.

**Table 1:** Model preparation and evaluation of RA

Type	Animal model	Modeling method	Evaluating indicators	Experimental animal	Typical references
Classical animal models	AIA model	Injected with 0.1 mL freshly prepared complete Freund's adjuvant (15 mg/mL)	Oxidative stress indicators (MDA, SOD, GSH, T-AOC, and NO) and inflammatory indicators (TNF-α and CO) in serum	Male SD rats	[145]
	CIA model	The collagen emulsion (0.2 mL) was injected into the rat-tail root. At day seven, another 0.1 mL of collagen emulsion was injected into the rat-tail root	Inflamed and swollen ankles and joints; Serum levels of TNF-α, IL-18; Osteopontin in serum, synovium, and cartilage; Synovial hyperplasia, inflammatory cell infiltration in synovium and the degree of cartilage degradation	Female SD rats	[129]
TCM syndrome induced animal models	Wind, damp, cold and heat exposure model	Rats'hind paws were put into water at a constant temperature of 45 ℃ and 4 ℃ respectively for 10 min and then rats were blown with wind at a temperature of 20 ℃ for 10 min twice a day for 14 days	Body-weight; Paw swelling; Blood cells analysis; Spleen and thymus coefficients; Autoantibodies and serum cytokine changes; Histopathology	Female SD rats	[191]
Syndrome-combined disease RA models	wind-cold-dampness syndrome CIA model	The right posterior foot claw, back, and tail root of the rats were injected with 0.1 mL emulsifier. Seven days later, the rats were immunized once with 0.1 mL emulsifier and stimulated by climate box with temperature 0–4 ℃, humidity 80–99%, wind speed 6 m/s, 30 min for 15 days	The degree of foot swelling; Pain threshold; AI score; Serum CRP, ESR, RF, TLR2; TLR4, and NF-κB in synovial tissue	Male SD rats	[19]
	Toxic dampness obstruction model	Collagen emulsion hypodermic (1 mg/mL) injected into the left foot of rats, and equivalently reinjected after 5 days. Administer of fat (1 g/100 g) daily and stimulated with RH (90 ± 4) % and T (24 ± 2) ℃ for 10 days	IL-6, TNF-α, VEGF in serum; Content of TGF-β1mRNA in periarticular soft tissue; The expression of AQP1, AQP2, AQP3 in serum and periarticular soft tissue; Activity of Na^+^, K^+^-ATP enzyme in spleen	Male Wistar rats	[192]
	AIA-M rat model	Injected intradermally at the base of tail with 10 mg/mL M tuberculosis H37 Ra suspended in Freund's complete adjuvant kept in the artificial climate box for 2 h daily with certain wind velocity (6 m/s), temperature (37 ℃) and humidity (90%) for a period of 15 days	The diameter of the limb, and arthritis score; Quantified data revealed that BMD, TMD, BV/TV ratio, and Tb.Th, BS/BV ratio and Tb.Sp	Male Lewis rats	[151]

The influence of dampness on RA is widely recognized. TCM syndrome RA models primarily consist of the wind-cold-dampness syndrome model and the wind-heat-dampness syndrome model. The wind-cold-dampness syndrome RA model involves the induction of an immune response using type II collagen and exposure to a wind (speed 6 m/s), humidity (80%−99%), and cold (0–4 °C) environment for 15 days, 30 min per day. This model resulted in reduced joint temperature; significant swelling of the foot; increased arthritis index (AI) score and pain threshold; and increased levels of CRP, ESR, and RF in the serum. Furthermore, it leads to increased expression of the TLR2, TLR4, and NF-κB proteins and mRNAs in knee joint tissue [128]. In addition, a wind-heat-dampness syndrome (RA) model was prepared using complete Freund's adjuvant combined with exposure to a wind (speed 6 m/s), humidity (90%), and heat (37 °C) environment for 15 days. This model exhibited increased joint temperature; elevated AI score and pain threshold; rough bone surface; severe bone erosion; inflammatory cell infiltration; synovial hyperplasia; bone destruction in the knee joint; immune-related visceral lesions; and increased lactate dehydrogenase activity as a cell death factor in serum [151]. The Dampness-Heat Obstruction symptom RA model combines spleen deficiency with dampness (a high-fat diet) in a humid environment and FA-related arthritis to induce changes in the AI score and inflammatory factor levels in serum and joint tissues [192]. These combined disease RA models exhibit disease characteristics and pathological changes consistent with the clinical manifestations and pathological changes observed in RA patients, indicating a departure from the limitations of traditional RA models.

## Nonalcoholic fatty liver disease and *Shi Zheng*

### Synopsis of nonalcoholic fatty liver disease

**Fig. 4:**
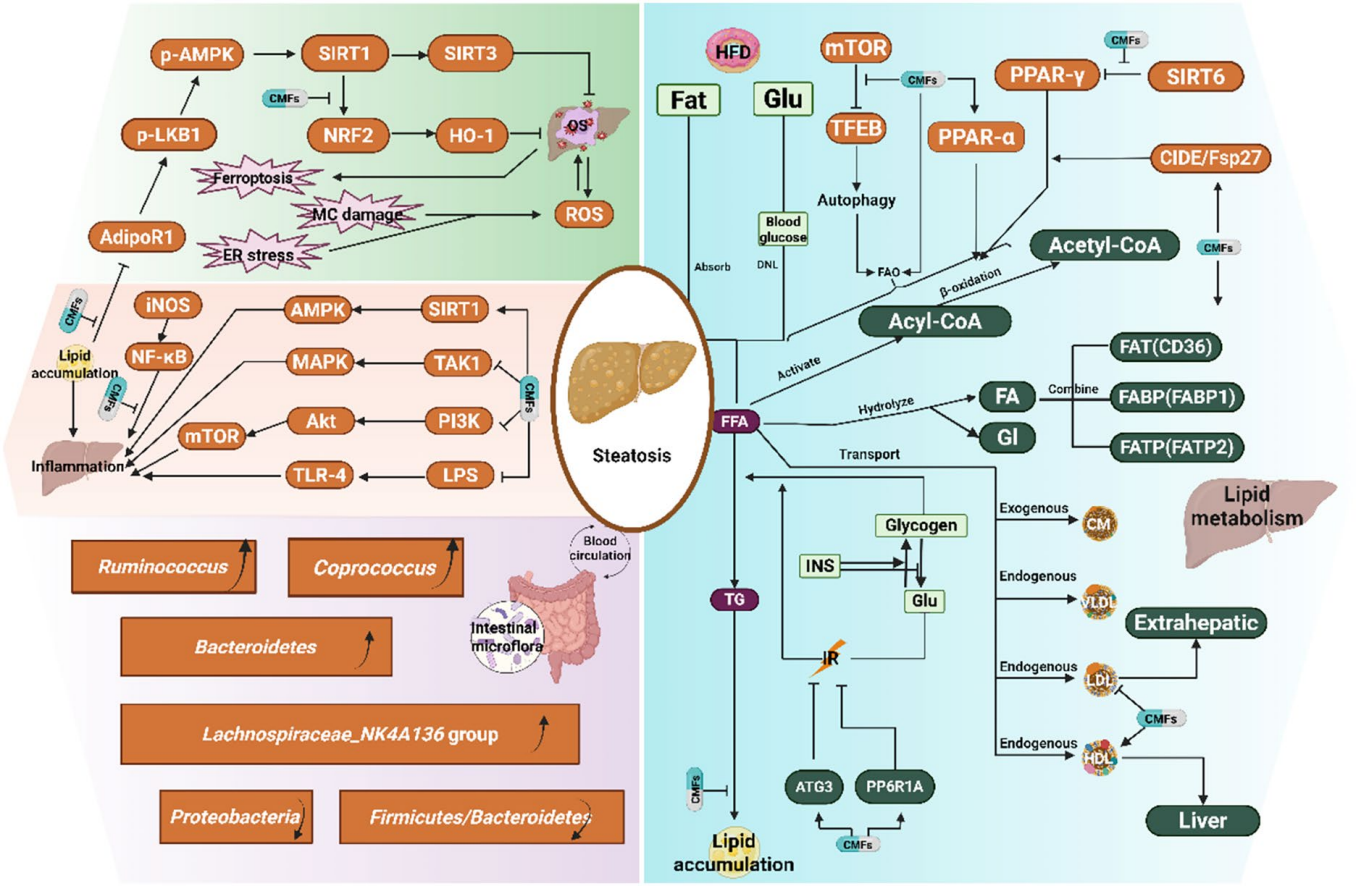
The mechanism of CMFs and their active substances for inhibition of NAFLD. *OS* Oxidative stress, *MC damage* mitochondria damage, *ER stress* endoplasmic reticulum stress, *Glu* glucose, *DNL* de novo synthesis, *FAO* fatty acid oxidation, *FFA* free fatty acid, *FA* fatty acid, *Gl* glycerol, *INS* insulin, *IR* insulin resistance, *TG* triglyceride, *CM* chylomicron, *VLDL* very low density lipoprotein, *LDL* low density lipoprotein, *HDL* high density lipoprotein

Nonalcoholic fatty liver disease (NAFLD) is a clinicopathological syndrome characterized by diffuse hepatocyte bullous fat induced by liver damage, excluding alcohol and other definite factors, including NAFLD and nonalcoholic steatohepatitis (NASH), whose incidence rate in modern adults has reached 25%. NASH patients with hepatic steatosis and chronic inflammation have a greater tendency to develop liver injury and end-stage liver diseases such as cirrhosis. Disorder of lipid metabolism is an important cause of NAFLD (Fig. 1), excessive accumulation of triglycerides in patients' liver cells can easily lead to oxidative stress and inflammation for a long time, which is an important risk factor leading to metabolic disorder and increasing the risk and mortality of malignant tumor, diabetes and coronary artery disease, among which the prevalence rate of NAFLD in diabetic population has reached 71% [193, 194]. Currently, there are no approved drugs for the treatment of NAFLD. Multiple hypoglycemic drugs have been evaluated for their efficacy in treating NAFLD due to the presence of insulin resistance in almost all NAFLD and diabetic patients. These include biguanides, glucagon-like peptide 1 receptor (GLP-1) agonists, PPAR agonists, and farnesol X receptor agonists such as metformin, liraglutide, and pioglitazone (Fig. 4) [195].

Due to its complexity and individual variability, the management of NAFLD necessitates a personalized therapeutic strategy [196]. TCM provides individualized management with favorable outcomes and unique advantages in the management of NAFLD. Modern research has shown that the expression of specific immune cells in patients with fatty liver with diverse syndromes exhibits significant discrepancies [197]. This includes the application of Gegen Qinlian decoction and JianPi-QingHua formula. In TCM, NAFLD is categorized as Ganpi syndrome, and treatments aimed at eliminating dampness are extensively employed due to the phlegm-dampness constitution, which is a potential risk factor for NAFLD [198]. The mechanisms underlying TCM treatments for NAFLD are becoming increasingly clear through in-depth investigations of the mechanisms of active ingredients. For instance, Potentilla discolor Bunge has been demonstrated to exert antioxidant and anti-inflammatory effects, improve lipid metabolism, and ameliorate insulin resistance in NAFLD [199]. Furthermore, various terpenoids derived from natural products have been shown to mitigate the pathological state of NAFLD [200].

### Lipid metabolism

The pathogenesis of fatty liver disease is associated with the accumulation of triglycerides (TGs) in the cytoplasm of hepatocytes, which results from an imbalance between lipid accumulation (uptake of free fatty acids (FFAs) and de novo lipogenesis) and clearance (mitochondrial fatty acid oxidation (FAO) and export via very-low-density lipoprotein (VLDL) particles). This imbalance is often induced by a high-calorie, high-fat, or high-fructose diet, which disrupts lipid metabolism in hepatocytes. A portion of FFAs enters the liver via the portal circulation, thereby augmenting both lipid synthesis and gluconeogenesis [201].

Dachaihu Decoction, Shenling Baizhu powder [202], JiGuCao capsule formula [203], Linghe granules [204] have been demonstrated to modulate hepatic lipid metabolism by reducing serum LDL and increasing HDL in NAFLD model rats [205]. Schisandrin B, a bioactive compound in *Schisandra chinensis* (Turcz.) Baill., exerts anti-steatotic effects by activating autophagy and promoting FAO via the AMP-activated protein kinase (AMPK)/mTOR pathway [206]. Isosilybin enhances FAO and inhibits lipid synthesis, thereby ameliorating hepatocyte steatosis [207]. Moreover, the overexpression of transcription factors associated with lipid metabolism, such as Rbbp4, Tcea1, and ILF2, has been shown to improve steatosis in NAFLD patients and may serve as potential biomarkers for the disease [208].

PPARs, including PPAR-α, PPAR-β/δ, and PPAR-γ, play crucial roles in regulating lipid metabolism, energy metabolism, inflammation, and fibrosis. PPAR-α is highly expressed in various organs and modulates liver fat accumulation by regulating the expression of genes related to lipoprotein metabolism, FAO, and cholesterol catabolism. Upregulation of PPAR-α enhances the β-oxidation activity of fatty acids and reduces hepatic TG levels. CMFs (such as Sijunzi, Lizhong, Fuzilizhong decoctions, and Qige Decoction [209]) and active ingredients (such as curcumin [210] and Oxymatrine [211] counteract the downregulation of PPAR-α associated with NAFLD [212]. PPAR-β/δ primarily regulates mitochondrial metabolism and fatty acid β-oxidation, and its agonists promote fat catabolism to ameliorate NAFLD. PPAR-γ influences biological processes such as adipocyte differentiation, lipogenesis, and lipid metabolism, and improves insulin resistance, inflammation, oxidative stress, ER stress, and fibrosis [213]. SIRT6 has been demonstrated to modulate the expression of fatty acid transporters by inhibiting PPAR-γ. Diosgenin ameliorates NAFLD by regulating the expression of SIRT6-related fatty acid transporters (decreasing the expression of CD36, FATP2, and FABP1) [214].

IR serves as a central driver of lipid metabolism disorder of NAFLD. The liver secretes various lipids and metabolites that function as signaling molecules, including lipoproteins, ketones, acylcarnitines, and bile acids, which in turn regulate insulin action. Hyperinsulinemia directly stimulates hepatic lipogenesis and lipid accumulation while indirectly suppressing hepatic glucose production [215]. Clinical studies have demonstrated that Lingguizhugan Decoction effectively ameliorates IR in overweight/obese NAFLD patients by modulating the DNA N6-methyladenine modification of protein phosphatase 1 regulatory subunit 3 A (PPP1R3A) and autophagy-related 3 (ATG3) [216]. The cell death-inducing DNA fragmentation factor-α-like effector (CIDE) family proteins plays a role in promoting the growth, fusion, and lipid storage of lipid droplets, including Cidea, Cideb, and Cidec/Fsp27, in hepatocytes and adipocytes. Banxia Xiexin Decoction, for instance, improves hepatic steatosis and IR induced by a high-fat diet by enhancing mitochondrial and peroxisomal fatty acid oxidation mediated by Cidea and Cidec [217]. Furthermore, Lactobacillus and Bifidobacterium exert cholesterol-lowering effects and contribute to the recovery of NAFLD patients. *Salvia miltiorrhiza* polysaccharide, when combined with Bifidobacterium bifidum V (BbV) and Lactobacillus plantarum X (LpX), refines the gut microbiota and improves IR in NAFLD mice [218].

### Oxidant stress

In patients with NAFLD, the excessive accumulation of lipids in hepatocytes can lead to oxidative stress and inflammation, thereby causing liver cell damage, apoptosis, and progression to hepatitis [219]. The accumulation of FFAs in hepatocytes and oxidative stress stimulate the production of reactive oxygen species (ROS) by mitochondria, the endoplasmic reticulum, and NADPH oxidase. This disrupts the balance between ROS production and antioxidant clearance, contributing to the pathogenesis of NAFLD [220]. Various plant extracts containing flavonoids, polyphenols, terpenoids, and alkaloids have been utilized to treat NAFLD and mitigate oxidative stress via multiple pathways [221]. The AMPK pathway is pivotal in maintaining the equilibrium between oxidation and antioxidation, and its activation by ROS helps restore cellular homeostasis. Atractylenolide III activates the AMPK/SIRT signaling pathway mediated by AdipoR1 in the liver, thereby protecting hepatocytes and ameliorating liver injury, lipid accumulation, oxidative stress, inflammation, and fibrosis in NAFLD mice [222].

Nuclear factor erythroid 2 related factor 2(Nrf2) is a redox-sensitive transcription factor that is released and translocated to the nucleus in response to excessive ROS. Nrf2 promotes the transcription of antioxidant genes and enhances the levels of antioxidant enzymes, thereby regulating the redox system. Extracts of Hedansanqi Tiaozhi Decoction, ginkgolide B, Pinocembrin [223], Oroxylin A [224] and Dihydrotanshinone I [225] protect hepatocytes by stimulating the Nrf2 pathway and mitigating iron-related cell death caused by oxidative stress [226, 227].

### Inflammation

NASH is a primary risk factor for liver fibrosis and cirrhosis, and its progression is influenced by oxidative stress and inflammation. Explained by the "two-hit hypothesis. "Inflammation in NASH is triggered by the systemic inflammatory environment created by inflammatory cytokines derived from adipose tissue in patients with NAFLD [228]. The hepatic inflammatory pathway is activated by bacterial translocation and the secretion of inflammatory cytokines and interferon due to an imbalance in intestinal ecology and dysfunction of the intestinal barrier [229]. Toll-like receptor 4 (TLR4) has been found to reduce the mRNA levels of TNF-α and IL-6 in an LPS-induced inflammatory model in RAW264.7 cells, thereby alleviating inflammation. It also mitigates NASH-related liver injury by inhibiting the TLR4 pathway [230]. Inflammation-mediated metabolic pathways such as AMPK and NF-κB are closely related to liver lipid metabolism and inflammation [231]. Huanglian-Hongqu herb pair treated NAFLD by targeting the NF-κB/NLRP3 pathway [232]. Besides, saikosaponin D functions as an anti-inflammatory agent and antioxidant in the treatment of NASH by inhibiting the gene expression of NF-κB and increasing the expression of antioxidant enzymes in the liver [233]. SIRT1 acts as a regulator of lipid metabolism and inflammation by inhibiting the AMPK and NF-κB pathways. The JianPi-QingHua formula intervenes in lipid accumulation and inflammatory reactions in NAFLD by activating SIRT1/AMPK signaling and attenuating the NF-κB pathway [234]. Cordycepin (3′‐deoxyadenosine) acts as an AMPK activator and mitigates metabolic stress-induced hepatic steatosis, inflammation, injury, and liver fibrosis [235]. TGF-β-activated kinase 1 (TAK1) regulates lipid metabolism as an upstream kinase of the NF-κB and MAPK pathways. Breviscapine, a TCM extract primarily containing baicalein, binds directly to TAK1 and inhibits its phosphorylation and subsequent cascade reactions within the MAPK signaling pathway [236]. Tanshinone IIA also inhibits MAPKs/NF-κB signaling pathway, thereby improves fatty degeneration of NAFLD [237]. The NLRP3 inflammasome plays a role in various inflammatory diseases, and active components of TCMs, such as rhubarb-free anthraquinone, echinatin, gentiopicroside, Ginger essential oil, Paeonol, and Panaxydol, inhibit the activation of the NLRP3 inflammasome to improve liver inflammation in NAFLD [238–244].

### Intestinal microflora

The intestinal barrier is a complex system, and the liver receives the majority of its blood supply from the intestine via the portal circulation, thereby establishing the "gut-liver axis."This axis facilitates the translocation of inflammation-related bacterial products and metabolites from the gut to the liver, exacerbating inflammation in NASH [245]. Patients with NAFLD commonly exhibit imbalances in their gut microbiota, which can be addressed with interventions such as spleen-strengthening and liver-draining formulas. One such formula has been shown to improve the relative abundance of *Coprococcus*, the *Lachnospiraceae_NK4A136* group, and the *Ruminococcus* genus [43]. Additionally, Simiaofang and Zexie-Baizhu Decoction  have been found to enhance hepatic lipid metabolism and reduce inflammation by increasing the relative abundance of *Akkermansia muciniphila* in the gut microbiota [246, 247].

Polysaccharides have demonstrated beneficial effects on abnormal lipid metabolism, inflammation, and oxidative stress in NAFLD through their capacity to modulate the abundance of the gut microbiota. Representative examples include *Astragalus polysaccharide*, *Poria polysaccharide*, and *Lycium barbarum polysaccharide*. These polysaccharides are primarily fermented by bacterial enzymes produced by colonic microorganisms, resulting in the generation of metabolites that are advantageous for regulating the gut microbiota and maintaining intestinal health [248]. *Astragalus polysaccharide* is particularly effective in correcting liver lipid metabolism, insulin resistance, oxidative stress, endoplasmic reticulum stress, inflammation, fibrosis, autophagy, and apoptosis in NAFLD patients. Its protective mechanisms are associated with the regulation of *Desulfovibrio vulgaris* and various signaling pathways, including SIRT1/PPARα/FGF21, PI3K/AKT/IRS-1, AMPK/ACC, mTOR/4EBP1/S6K1, GRP78/IRE1/JNK, AMPK/PGC-1α/NRF1, TLR4/MyD88/NF-κB, and TGF-β/Smad [249, 250].

Pachyman has shown promise in preventing the progression of NASH by maintaining intestinal microflora homeostasis and downregulating the NF-κB/CCL3/CCR1 axis [251]. Conversely, Lycium barbarum polysaccharide can regulate the abundance of *Bacteroidetes*, short-chain fatty acids (SCFAs), and *Proteobacteria* as well as the *Firmicutes/Bacteroidetes* ratio in the intestine to improve NAFLD [252]. SCFAs, which are the principal metabolic byproducts of intestinal microbial fermentation, are beneficial for enhancing glucose and lipid metabolism. Zanthoxylum bungeanum amides derived from *Zanthoxylum bungeanum* Maxim. (Rutaceae) have been shown to reduce weight, reverse liver lesions and fat accumulation, and improve oxidative stress in the liver tissue of high-fat diet-induced obese mice by increasing the abundance of high-yield SCFA-producing bacteria [253].

### Animal model

Primary NAFLD animal models include dietary-induced models, such as the high-fat diet, the high-fat/high-cholesterol diet, the methionine and choline deficiency diet, and the high-fat/high-sucrose diet; drug-induced models, including streptozotocin, CCl4, LPS, and tetracycline; disease-prone models, like hepatocyte- and macrophage-specific SREBP-1a knockout mice and genetically obese (ob/ob) mice; and spontaneous models, for example, ApoE mice [236, 246, 254–256]. The diet-induced animal model shares similarities with the concept of *Shi Zheng* in TCM, which involves improper diet. However, traditional disease and TCM syndrome combination models have not been sufficiently explored. The diet-induced animal model has the potential to induce NAFLD, expedite the modeling process, and reflect the disease process of liver damage caused by "*Shi Zheng*", which results from long-term overconsumption of fatty, sweet, and greasy products, as exemplified by the NAFLD model induced by a high-fat diet in ob/ob mice (Table 2) [257].

**Table 2:** Model preparation and evaluation of NAFLD

Type	Animal model	Modeling method	Evaluating indicators	Experimental animal	Typical references
Classical animal models	HFHC model	Protein, 14%; fat, 42%; carbohydrates, 44%; cholesterol, 2% diet for 16 weeks	Lipid accumulation; Inflammatory cell infiltration; liver injury, and fibrosis	Male C57BL/6 J mice	[236]
	HFHS model	High-fat diet and 30% (g/mL) sucrose solution	Glucose homeostasis; Serum AST, TG, TC, ALT, LDL-c and HDL-c concentrations	Male C57BL/6 mice	[246]
Susceptible animal model	Albumin-Cre/SREBP-1α flox and LysM-Cre/SREBP-1α flox mice	Albumin-Cre/SREBP-1α flox and LysM-Cre/SREBP-1α flox mice fed with an MCD diet containing 10.2% fat, 17.9% protein, and 57% carbohydrates for 10 weeks	Liver pathological changes	Albumin-Cre/SREBP-1α flox and LysM-Cre/SREBP-1α flox mice	[254]
	ob/ob mice	Fed with the normal diet	Fed with the normal diet, The hepatic TG and TC. The hepatic TG and TC. Hepatic fat accumulation	Male ob/ob mice	[255]
Spontaneous animal model	ApoE mice	Fed with the normal diet	AST and ALT. serum lipid profile (TC, TG and LDL-C)	ApoE-/-mice	[256]

## Gouty arthritis and *Shi Zheng*

### Synopsis of gouty arthritis

**Fig. 5:**
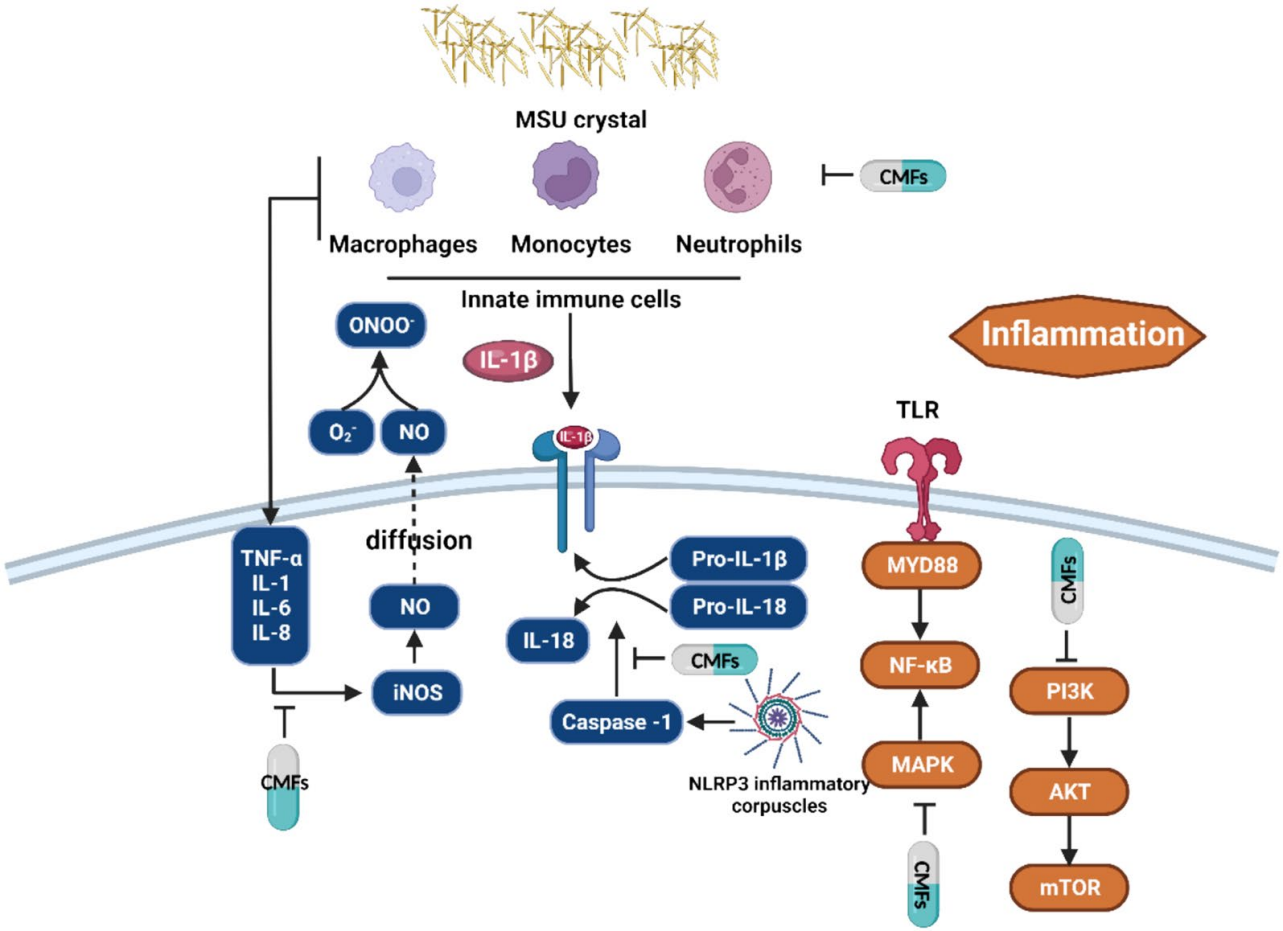
The mechanism of CMFs and their active substances for inhibition of inflammation in GA

Gouty arthritis (GA) is categorized as primary gout or secondary gout. Its prevalence in Asia, Europe, and the Americas ranges from 0.68 to 3.9%, with a male-to-female ratio of approximately 8:1 [258]. The significant increase in GA incidence is closely linked to modern lifestyles, particularly the consumption of alcohol and seafood. Hyperuricemia, which results from low uric acid excretion or purine metabolism disorders, together with the deposition of monosodium urate (MSU) crystals, can lead to joint inflammation characterized by redness, swelling, increased skin temperature, pain, and limited joint mobility (Fig. 1). It is important to note that while hyperuricemia is a key indicator of GA, it is not the sole diagnostic factor. Dual-energy CT scans can be used clinically to detect MSU crystals in joints, aiding in the diagnosis of GA [259]. Urate deposits (tophi), chronic GA, and structural joint damage are long-term complications that affect GA patients, resulting in recurrent episodes of the disease [258]. Importantly, disturbances in the intestinal microbiota and metabolism in GA patients may serve as intrinsic triggers for urate degradation disorders and inflammation (Fig. 5) [260, 261].

Colchicine, NSAIDs, and glucocorticoids are frequently employed to alleviate inflammation and severe pain during the acute phase of GA. However, these medications can induce serious adverse reactions, including gastrointestinal ulcers, hepatic and renal dysfunction, neurotoxicity, and immunosuppression. In the intermittent or chronic phase of GA, medications aimed at inhibiting uric acid production (such as allopurinol), promoting uric acid excretion (such as probenecid and sulfinpyrazone), and maintaining urine pH (such as sodium bicarbonate tablets) are typically prescribed [262]. Nonetheless, these drugs have been associated with severe and potentially life-threatening adverse events, including allopurinol-induced hypersensitivity syndrome [263]. Therefore, there is an urgent need for more accurate, rapid, long-term, and safe treatment options for GA.

TCMs have demonstrated efficacy in enhancing the cure rate for GA when used in conjunction with conventional therapies. Specifically, variants of the classically effective CMF Gout Prescription, when combined with etoricoxib and benzbromarone tablets, have been used to treat acute GA patients with dampness-heat syndrome, achieving an effective treatment rate of 95.45% [264]. Additionally, clinical trials have highlighted the significant efficacy and minimal adverse events of tongfengding capsules, tongfengtai granules, and simiao powder, suggesting their potential as promising treatment strategies for GA [265–268]. According to TCM theory, factors such as dampness, heat, toxic pathogens, phlegm-dampness, dampness-heat, and blood stasis are relevant to GA, with phlegm dampness, dampness heat, and blood stasis constitutions accounting for approximately 35–68% of GA patients [259, 269]. Consequently, GA is considered a major *Shi Zheng* disease, and the therapeutic mechanisms of CMFs and their active ingredients are being studied. Modern pharmacological research has revealed the mechanisms of CMFs and active ingredients in treating GA, including the alleviation of inflammation, regulation of UA levels, modulation of small-molecule metabolites, and improvement of the gut microbiota to address UA levels and related metabolic pathway disorders in hyperuricemia, including tryptophan metabolism, arginine biosynthesis, purine metabolism, arginine and proline metabolism, beta-alanine metabolism, the citrate cycle (TCA cycle), glycerophospholipid metabolism, and linoleic acid metabolism. Effective CMFs for GA, such as Sanmiao pills and Simiao pills, have been developed based on Ermiao pills [270].

### Inflammation

GA inflammation is typically caused by stimulation with MSU crystals. After inflammation occurs, synovial cells, mononuclear macrophages, and neutrophils release inflammatory particles and proinflammatory factors. Evidence suggests that the Shuang-Qi gout capsule can reduce the release of proinflammatory factors, such as TNF-α and IL-1β, thereby attenuating inflammation in GA. *Atractylodes lancea* (Thunb.) DC has been found to improve GA inflammation by modulating inflammatory factors and pathways related to apoptosis, including TNF-α, IL-6, IL-1β, prostaglandin endoperoxide synthase 2 (PTGS2), MAPK14, and NF-κB p65 (RELA) [271–274].

The nucleotide oligomerization domain (NOD)-like receptor (NLR), a cytoplasmic receptor, plays a crucial role in innate immunity by recognizing pathogen-related molecular patterns and damage-related molecular models [275]. Miaofuzhitong granules ameliorate GA by regulating the NLR pathway. Berberine, 3-feruloylquinic acid, pellodendrine, neoastilbin, chlorogenic acid derivatives, and isoacteoside have been identified as the main active ingredients in Miaofuzhitong granules [276]. Inflammasomes, which belong to the NLR family, participate in protein synthesis and promote the secretion of IL-1β and IL-18, inducing inflammation. Cinnamomum cassia has been shown to alleviate GA-related inflammation by inhibiting the activation of inflammatory corpuscles, such as NLRP3, NLRC4, and AIM2 [277]. Furthermore, caspase-1, a key component of inflammasomes, can be inhibited by coptisine, which may be a key mechanism for GA induced by NLRP3 inflammasomes [278]. Classical CMFs (Wuwei Xiaodu Drink), Modified CMFs (modified Sanmiao pills) and effective components of TCMs (procyanidins B2 and tetrahydropalmatine) have also been found to alleviate GA by inhibiting the activation of NLRP3 inflammatory corpuscles [279–282].

NF-κB is a key inflammatory pathway, the activation of TLR/MyD88/NF-κB cascade is a critical pathway in the pathogenesis and treatment of GA. TLR can specifically recognize sodium urate (MSU) and mediate its signal transduction, thus regulating inflammatory response. This pathway has been shown to be involved in the mechanisms of action of modified Sanmiao pills, Jiaweisimiao pills, isovitexin, and β-caryophyllene in the treatment of GA [281, 283, 284]. Upon pathogen recognition by TLRs, an innate immune response is induced, leading to an inflammatory reaction mediated by the recruitment of MyD88 and the production of pro-IL-1β stimulated by NF-κB. Guizhishaoyaozhimu Decoction, Yinhua Gout granules and Baihu Guizhi Decoction, the effective Chinese medicine formulas, have been found to inhibit NF-κB and shows promise in treating GA [285–287]. Additionally, MAPK, PI3K, AKT, and NLRP3 also can regulate the expression of the NF-κB signaling pathway [40]. Baeckea frutescens L. extract, specifically baeckein E, has been found to inhibit the activation of NLRP3 inflammatory corpuscles in lipopolysaccharide (LPS)-induced macrophages and gout mouse models by blocking the MAPK/NF-κB signaling pathway and mitigating oxidative stress induced by mitochondrial damage [288]. Kaempferol, a natural flavonoid, also improves GA by mediating NLRP3/NF-κB pathway [289]. Moreover, elevated expression of the Cyr61 protein has been implicated in the pathogenesis of GA, inducing synovial cells to produce inflammatory cytokines such as IL-1β, TNF-α, and IL-6, which are partially dependent on NF-κB [290].

Furthermore, the Huangqin Qingrechubi capsule has been shown to counteract the inhibitory effect of lncRNA H19 overexpression on the vitality of FLSs and the adiponectin pathway while suppressing the PI3K/AKT pathway [291]. Simiao has demonstrated anti-inflammatory effects in the treatment of GA by promoting M3 polarization and inhibiting the PI3K/AKT pathway [292]. Autophagy is induced in response to inflammation as a form of cellular self-protection, and the classical pathway regulating autophagy involves the PI3K/AKT/mTOR pathway. Qingre Huazhuo Jiangsuan decoction and ZeXie decoction  have been found to inhibit the PI3K/AKT/mTOR pathway, thereby leading to reduced expression of inflammatory factors and UA levels in GA patients [293, 294].

Immune cell subset imbalances involving macrophages and neutrophils can exacerbate inflammation in GA [295]. The therapeutic mechanism of action of the Shuang-Qi gout capsule in treating GA is associated with the promotion of neutrophil death, specifically through neutrophil extracellular traps (NETs) and NETosis [296]. Similarly, Shirebi granules alleviated joint redness based on the inhibition of NETs expression [297]. Additionally, lipid metabolism is closely linked to acute GA-related inflammation. The prevalence of lipid metabolism disorders, including elevated total cholesterol (TC), triglyceride (TG), and low-density lipoprotein cholesterol (LDL-C), as well as reduced high-density lipoprotein cholesterol (HDL-C), in GA patients has reached 72.73%. These lipid abnormalities may contribute to immune dysregulation and inflammation [298]. Inflammation can also be induced by a deficiency of SCFAs [260]. Moreover, an imbalance in the intestinal microbiota is strongly associated with GA-related inflammation, and the modified Baihu decoction has been shown thereby alleviating the abundance of various bacterial families, including *Lachnospiraceae*, *Muribaculaceae*, *Bifidobacteriaceae*, Lactobacillaceae, *Erysipelotrichaceae*, *Ruminococcaceae*, *Prevotellaceae*, and *Enterobacteriaceae*, to treat GA-related inflammation [299].

### UA production and excretion

The strong correlation between UA levels and GA has been widely recognized, and the production and excretion of UA are vital for the body's regulation of UA levels. The primary sources of UA are diet (approximately one-third) and endogenous metabolism, with the kidneys and gastrointestinal tract being the main routes of excretion. The kidneys play a crucial role in UA excretion and are regulated by urate transporters such as GLUT9, urate transporter 1 (URAT1), and ABCG2. When UA reaches 6.8 mg/dL, it can crystallize and form MSU crystals, which activate the NLRP3 inflammasomes to produce IL-1β, leading to increased serum UA concentrations, tophus formation, and inflammation in GA [300].

Xanthine oxidase (XOD) is a critical enzyme involved in UA production, and overexpression of XOD predisposes individuals to excessive UA production. The Chuanhu anti-gout mixture can reduce plasma UA levels by decreasing XOD expression in the liver and URAT1 expression in the kidneys [301]. Additionally, various active components found in TCMs inhibit UA synthesis and promote UA excretion by inhibiting hepatic XOD activity and regulating renal UA transporters. Representative of these compounds include dihydroberberine and berberine in *Phellodendri Chinese* Cortex; apigenin 7-O-glucoside in the *Paeonia* × *suffruticosa* Andrews leaf; and smilaxchinoside A, smilaxchinoside C, ripidroside B, and timosaponin J in the roots and rhizomes of Smilax riparia, as well as TCM formulas comprise Sanmiao wan and Juanbilijieqing fang [302–308].

**Fig. 6:**
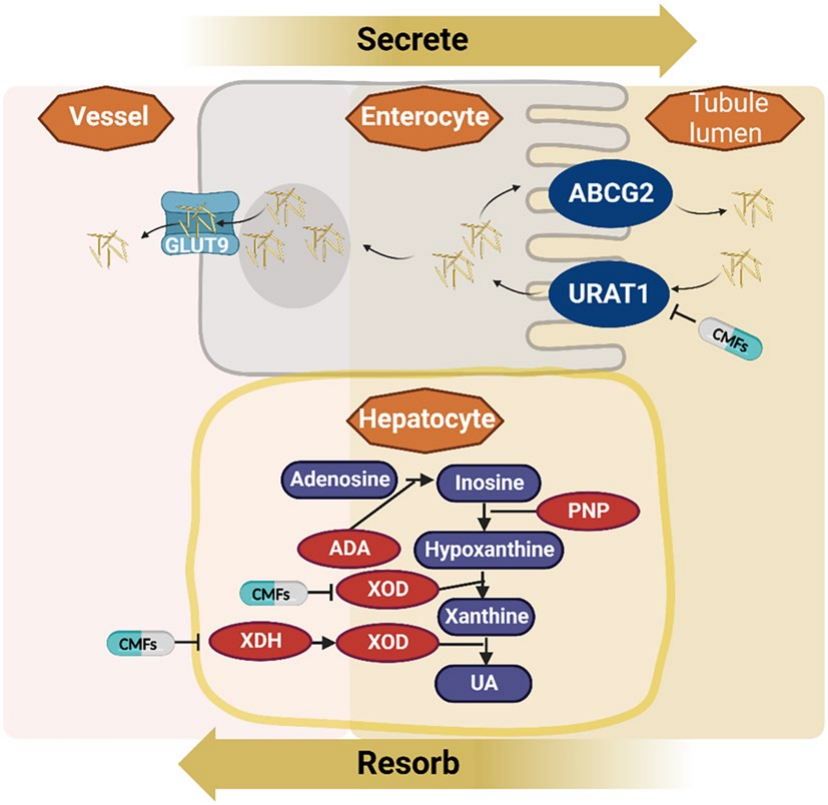
The mechanism of CMFs and their active substances for regulating UA production and excretion in GA

Increasing UA excretion is a reliable approach for treating gout, and emodin has been shown to increase UA excretion in hyperuricemic rats [309]. Furthermore, Shuang-Qi gout capsules promote UA excretion and protect renal function by regulating the expression and mRNA levels of renal transporters [310]. The Xie-Zhuo-Chu-Bi-Fang formula increases the expression of miR-34a, which inhibits URAT1 mRNA, thereby reducing UA levels in hyperuricemia [311]. It is well established that UA is a metabolite of purine. The Er-Miao-Wan formula and Tongfengtang powder improve UA levels by modulating purine metabolism through various mechanisms [312, 313]. Generally, UA in the bloodstream is transported to the intestinal cavity by UA transporters in intestinal epithelial cells. Therefore, the metabolism of purine and UA is regulated by the gastrointestinal microbiota [314]. Additionally, a deficiency in *Enterobacteriaceae* may hinder UA degradation [260]. Further elucidation of the direct relationship between TCMs and the intestinal microbiota, as well as between the production and excretion of UA is warranted, given the significant influence of the intestinal flora on the pathogenesis and treatment of GA (Fig. 6).

### Animal model

When investigating the underlying mechanisms of GA, researchers primarily utilize genetic induction and environmental induction models, with rats, mice, rabbits, and chickens serving as experimental animals [315]. Overseas pharmacological studies on GA predominantly employ genetically modified, drug-induced, or small-molecule inhibitor-induced animal models, as these models induce pathological characteristics associated with hyperuricemia, inflammation, and tophus formation. However, these models have limitations in studying the mechanism of action of CMFs in GA due to their failure to reflect the systematic nature of TCM theory. Importantly, these models do not fully encompass fundamental processes involved in disease occurrence and development in the body, such as a high-fat and high-sugar diet, a high-purine diet, alcohol consumption, or hot and humid environments. Chronic consumption of diets high in sugar, fat, and protein can induce abnormal UA metabolism and chronic inflammation, while ethanol significantly increases urate synthesis, thereby elevating the risk of GA [316, 317]. Furthermore, environmental factors, including season, are closely associated with GA, with urate concentrations in the body peaking during the summer months [318].

A GA model is induced solely by environmental factors, specifically by a hot and humid environment with a remarkably low  modeling rate. Therefore, to study the treatment of damp-heat syndrome (GA) with CMF, a drug-induced model of combined damp-heat syndrome (GA) was utilized. The damp-heat syndrome (DHS) GA model was established by combining joint inflammation induced by the injection of microcrystalline sodium urate solution with a damp-heat syndrome background. This background is created through the administration of honey, alternate perfusion with fat or liquor, and integration with an artificial climate box maintaining high temperature (32 ± 2 ℃) and high humidity (92 ± 3%) [319]. In the pharmacological study of Tongfeng Qingxiao formula on GA, the animal model of GA with damp-heat syndrome was applied to explore the evaluation indexes of TCM symptoms [320]. However, this modeling method does not significantly enhance the presence of UA in serum and is therefore unsuitable for studying the effects of CMFs on concurrent hyperuricemia and inflammation. Hyperuricemia animal models induced by potassium oxonate or hyperpurine diets (hypoxanthine, yeast extract, and potassium oxalate) could be applied to the GA model with DHS to improve the applicability of the disease-syndrome combination model in GA research [321, 322].

In addition, rodents are the primary experimental animals used in GA research, although they have certain limitations. Rodents metabolize purines into allantoin, whereas in humans and birds, purines are metabolized into urate due to the lack of uricase. Therefore, birds fed a high-protein or high-purine diet are more likely to establish hyperuricemic models, making them suitable for the study of GA in individuals with hyperuricemia (Table 3) [323, 324].

**Table 3:** Model preparation and evaluation of GA

Type	Animal model	Modeling method	Evaluating indicators	Experimental animal	Typical references
Classical animal models	PO-induced hyperuricemia mice model	Administrate PO (12.5 mg/mL)	Serum uric acid levels; Urinary uric acid level; IL-1β, IL-6, and TNF-α	Kun-Ming strain of mice	[321]
	MSU crystal-induced inflammation rat model	Hypodermic injection of 0.1 mL (10 mg) of endotoxin free MSU crystal suspension into the right foot pad			
	Hyperuricemia mice model	Received 200 μL of high purine solution for 8 weeks by gavage, which contained hypoxanthine (200 mg kg^−1^ d^−1^), yeast extract (30 mg kg^−1^ d^−1^) and potassium oxalate (200 mg kg^−1^ d^−1^)	The indexes of the liver, kidneys and spleen; Serum uric acid, urea nitrogen and creatinine; Urate absorptive transporters (OAT4, GLUT9 and URAT1) and urate secretory transporters (ABCG2, MRP4 and OAT1)	ICR mice	[322]
Syndrome-combined disease GA models	GA model with Dampness-heat syndrome	Injection of MSU solution, and administration of honey, alternate perfusion with fat or liquor, and integration with an artificial climate box maintaining high temperature (32 ± 2 ℃) and high humidity (92 ± 3%)	Urine aquaporin 2, ET	SD rats	[319]

## Other diseases and *Shi Zheng*

### Jaundice

**Fig. 7:**
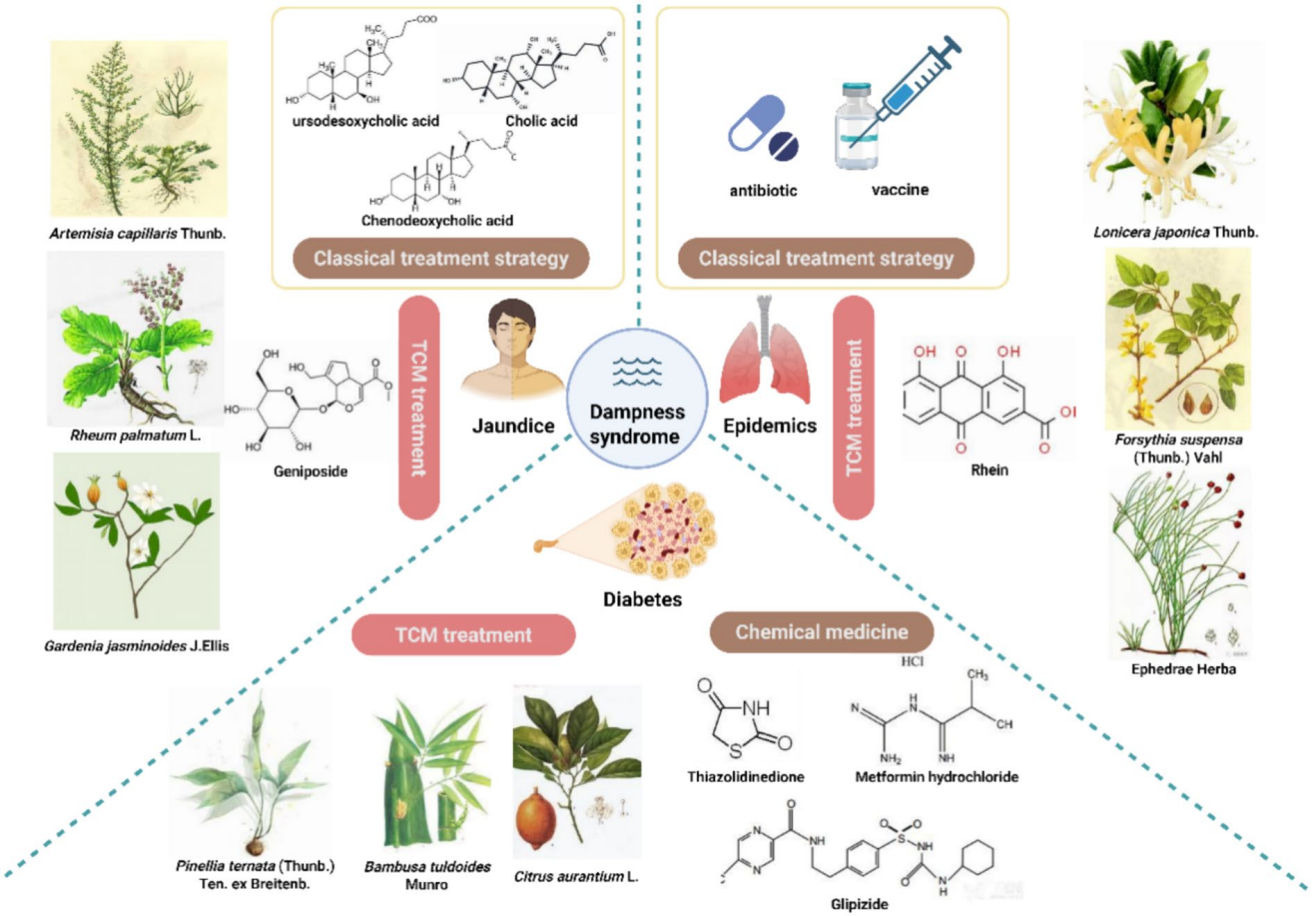
The treatment of CMFs and classical treatment for jaundice, epidemic and diabetes

Jaundice is characterized by an imbalance in bilirubin metabolism, resulting in an excessive concentration of bilirubin in the bloodstream and yellow discoloration of the sclera, mucosa, skin, and other tissues. The clinical manifestations primarily include hemolytic jaundice, hepatocellular jaundice, and obstructive jaundice. Currently, clinical treatments for jaundice involve the use of drugs or surgical interventions [325]. Ursodeoxycholic acid, cholic acid, and chenodeoxycholic acid are frequently employed in the treatment of jaundice. However, jaundice is often a complication of other diseases, and patients typically require combined medications to address complex disease systems. This approach tends to increase the risk of adverse reactions. Many CMFs can treat jaundice and protect the liver through their superior multitarget effects. Examples of such CMFs include Zhizi Dahuang decoction, Yinchenhao decoction, and Dahuang Xiaoshi decoction [326]. TCM identifies dampness, heat, cold, blood stasis, and spleen deficiency as the pathogenesis of jaundice. Damp pathogens are considered the main cause of jaundice, and eliminating damp-heat CMFs are used to treat it [327]. The Jigucao capsule has been shown to have a positive effect on damp-heat jaundice by regulating biomarkers such as arachidonic acid, phenylpyruvic acid, and L-urobilin [328]. Additionally, geniposide, a quality marker of Yinchenhao decoction, could be a potential active ingredient in the treatment of damp-heat jaundice (Fig. 7) [329].

### Epidemics

Respiratory diseases such as H1N1, SARS, and COVID-19 have emerged globally with rapid spread, uncertain and complex early stages of development [330]. Treating epidemics involves reusing existing antiviral drugs, developing targeted antiviral drugs, and creating vaccines [331]. However, the research and development of new drugs can be time-consuming, potentially leading to larger-scale outbreaks.

TCM has long been recognized as a reliable approach for treating epidemic diseases. In TCM, epidemic diseases such as COVID-19 are classified as "epidemic dampness"with damp-heat syndrome or cold-dampness syndrome [332]. TCM intervention significantly reduced the severity and mortality rates of COVID-19 during the early stages of the outbreak, even without specific therapeutic drugs. These findings highlight the potential advantages of TCM. Additionally, three TCM drugs and three herbal formulas (3-drugs-3-formulas) have been validated as effective and safe based on long-term clinical evidence, particularly for treating sequelae following negative nucleic acid tests [333, 334]. Generally, TCM attenuates infection-related processes (cytokine storm, immune abnormality, coagulation abnormality) by inhibiting the replication and transcription of SARS-CoV-2 and interfering with the virus's normal physiological functions (Fig. 7) [335].

### Diabetes

Diabetes is a disease with an extremely high incidence rate, and 90–95% of cases are classified as Type 2 diabetes mellitus (T2DM), characterized by a progressive loss of insulin secretion in β-cells following the development of insulin resistance. This condition is often complicated by microvascular and chronic nephropathy [336]. Although drugs such as biguanides, sulfonylureas, thiazolidinediones, and insulin are effective in controlling blood glucose levels in T2DM patients, they also increase the risk of gastrointestinal adverse reactions, hypoglycemia, and cardiovascular diseases [337]. The diagnosis and treatment of diabetes via TCM are more comprehensive and focused, aiming to achieve optimal therapeutic outcomes. TCM identifies various syndromes associated with diabetes, including phlegm (dampness) and heat accumulation, heat-impaired fluid, deficiency of qi and yin, yin deficiency of liver and kidney, and deficiency of yin and yang phlegm-dampness obstructive type T2DM [338]. TCM refers to diabetes as "xiaoke"due to symptoms of excessive thirst and emaciation. Moreover, TCM recognizes dampness-heat syndrome as the primary cause of diabetes, with a mechanism involving heat and blood stasis induced by phlegm dampness [339, 340]. Treatment of damp-heat-induced T2DM patients typically involves strengthening the spleen and stomach while clearing heat and dampness using CMFs. These CMFs exhibit anti-inflammatory, antioxidative, blood lipid-regulating, glucose-lowering, and intestinal microflora-regulating effects [341]. For example, Wendan decoction has been shown to improve multiple diabetes indicators, such as BMI, fasting insulin, and blood lipids, through pathways associated with oxidative stress and inflammation (Fig. 7).

## Discussion

*Shi Zheng* is a prevalent TCM syndrome in regions south of the Five Ridges in China, primarily attributed to factors such as spleen deficiency and environmental influences. It can manifest independently or in combination with other syndromes, leading to pathological changes in organs and tissues, including the spleen, stomach, bones, and kidneys. These changes result in conditions such as diarrhea, abdominal pain, rheumatoid arthritis, and IgA nephropathy [342]. Therefore, the pathological characteristics and triggers of *Shi Zheng* exhibit pronounced heterogeneity and complexity. To date, only a handful of studies have systematically compared altered micro-molecular metabolite profiles and proteomic signatures between *Shi Zheng* patients and healthy controls, thereby delineating the distinctive molecular phenotypes of *Shi Zheng* [343]. Consequently, these investigations have yielded a panel of translational biomarkers and evidence-based diagnostic–therapeutic frameworks for managing *Shi Zheng* in clinical practice. Nevertheless, the mechanistic distinctions between endogenous and exogenous dampness manifestations within *Shi Zheng*, as well as the pivotal determinants governing multi-organ tropism, remain incompletely elucidated. Therefore, further systematic studies are warranted to systematically delineate the occurrence and development of *Shi Zheng*.

TCM formulas (CMFs) that address symptoms such as fever, dampness, yang deficiency, and spleen dysfunction have proven effective and are widely used to treat *Shi Zheng*-related diseases. However, due to the unclear relationship between TCM syndromes and modern diseases, CMFs are often used as a complementary therapy alongside modern drugs. Therefore, further investigation into the connections between *Shi Zheng* and modern diseases is essential. This includes categorizing diseases related to *Shi Zheng*, identifying their associated TCM syndrome patterns, and exploring existing models for disease combinations. Through these efforts, the mechanisms by which CMFs treat diseases by improving TCM syndromes can be elucidated. Although RA, NAFLD, and GA are classified under *Shi Zheng*, their pathological characteristics and pathogenesis differ significantly due to the influence of dampness. Modern research could aim to differentiate the TCM syndromes of these diseases through multi-omics analysis and molecular biological means to characterize the unique biology profile of *Shi Zheng* and validate the rationality of TCM syndrome diagnosis.

RA is primarily induced by external factors such as exogenous cold-damps or wind-damps, leading to inflammation, angiogenesis, and bone tissue damage. Conversely, NAFLD is caused by internal dampness resulting from spleen deficiency, which in turn leads to lipid metabolism disorders, oxidative stress, and inflammation. GA is induced by both external and internal dampness, resulting in disorders of UA metabolism and inflammation. Overall, *Shi Zheng* manifests as inflammation and metabolic disorders, eliciting various pathological reactions with different backgrounds. TCM treatments have demonstrated promising efficacy and fewer adverse reactions compared to conventional drugs in the management of diseases caused by *Shi Zheng*, likely attributable to their focus on syndrome diagnosis and personalized therapy. Therefore, it is crucial for traditional CMFs to emphasize their dampness-eliminating properties and incorporate the characteristics and key factors of TCM syndrome, thus highlighting the advantages of TCM treatment.

Various animal models can be utilized to simulate *Shi Zheng*, including inducing conditions through dietary manipulation involving high fat, high sugar, high protein, and high purine contents or by creating a controlled humid environment using an artificial climate box. Additionally, despite their low incidence and prolonged duration, TCM syndrome models for specific diseases can be combined with conventional disease models to refine their limitations and incorporate TCM syndrome factors. The immune-mediated type of RA model was combined with the TCM syndrome of external and internal dampness to create the syndrome-combined disease RA model, which is widely utilized in the study of CMFs for RA treatment. In rats and mice, FA and type II collagen are the primary immune inducers. To establish an external dampness syndrome model, the theory of dampness caused by spleen deficiency in TCM and stimulation from an artificial climate box were considered, resulting in a spleen deficiency animal model. Additionally, *Shi Zheng* can be induced in animal models by both internal and external dampness. The syndrome-combined disease RA models effectively mimic the immune response in the early stages of RA and correspond to TCM syndromes of CMF adaptation. These models are therefore appropriate for studying the mechanisms of CMFs in treating RA with various TCM syndromes.

The triggers of *Shi Zheng* in model preparation are relatively clear in current experimental paradigms; however, the precision of modeling parameters and the potential induction of complications remain insufficiently supported by experimental evidence. Systematic research on key parameters in animal models is therefore required to minimise false-positive outcomes. Taking the syndrome-combined disease RA model as an exemplar, investigators typically employ an artificial climatic chamber to deliver controlled environmental stimuli including wind, cold, heat and dampness, to induce TCM syndrome. Yet sustained exposure to damp-heat conditions can precipitate secondary infections [344], thereby compromising the specificity of the animal model and potentially undermining the validity of the results. Future studies should systematically investigate parametric settings, including temperature, humidity, wind velocity and the frequency of environmental exposure, and comprehensive histopathological profiling of susceptible tissues in this research will contribute to clarifying the potential relationship between exposure–response and organ-selectivity. Moreover, since the initiation of adaptive immunity constitutes a pivotal phase in RA pathogenesis, and TCM syndrome models that rely solely on environmental stimuli are intrinsically limited in triggering rapid and robust immune activation, immunostimulants such as complete Freund’s adjuvant are therefore warranted to recapitulate the local inflammatory milieu. Immune induction methods suitable for symptoms should be selected based on their applicability to TCM syndromes. Analyzing the differences in biomarkers between the RA model and the syndrome-combined disease RA model is crucial for elucidating the key factors of TCM symptoms through multiple approaches, which can reveal the microscopic characteristics of TCM symptoms. Therefore, further research should investigate *Shi Zheng* and other TCM symptoms to scientifically explain the rationality of TCM syndromes and the effectiveness of TCM in treating diseases.

## Conclusion

The modern diet, characterized by high-fat and high-protein intake, along with excessive alcohol consumption and humid environments in certain regions, has been associated with an increase in the prevalence of *Shi Zheng* and the incidence of diseases related to *Shi Zheng* [345–348]. These findings suggest that *Shi Zheng* may serve as a potential risk factor for these diseases [345, 349]. This review clarifies the advantages of TCM in treating these diseases by summarizing the effects and mechanisms of TCM on RA, NAFLD, and GA, all of which are related to *Shi Zheng*. In particular, TCMs have shown efficacy in the early stages of these diseases, such as autoimmune and inflammatory conditions in pre-RA, hyperuricemia in pre-GA, and insulin resistance in early NAFLD. Furthermore, as a risk factor, *Shi Zheng* can be diagnosed and treated before disease progression to improve patient prognosis. Therefore, this review summarizes the pathological characteristics of *Shi Zheng*, as well as the effective CMFs and active components for related diseases, providing a theoretical foundation for further research on TCM syndromes and the development of treatment strategies for these conditions.

## Data Availability

No datasets were generated or analysed during the current study.

## Abbreviations

ACPAs: Anti-citrulline protein antibodies
ADAMTSs: A disconnect and metalloproteinase with thrombospondin-like motifs
AI: Arthritis index
AMPK: AMP-activated protein kinase
ATG3: Autophagy-related 3
BbV: Bifidobacterium bifidum V
CHD: Coronary heart disease
CIDE: Cell death-inducing DNA fragmentation factor-α-like effector
c-JNK: C-Jun N-terminal kinase
CMFs: Chinese medicine formulas
CTGF: Connective tissue growth factor
DCs: Dendritic cells
DHS: Damp-heat syndrome
ERK: Extracellular signal-regulated kinase
FA: Freund's adjuvant
FAO: Fatty acid oxidation
FFAs: Free fatty acids
FLSs: Fibroblast-like synoviocytes
GA: Gouty arthritis
GLP-1: Glucagon-like peptide 1 receptor
HDL-C: High-density lipoprotein cholesterol
JAK/STAT: Janus kinase/signal transducers and activators of transcription
LDL-C: Low-density lipoprotein cholesterol
LPS: Lipopolysaccharide
LpX: Lactobacillus plantarum X
MAPK: Mitogen-activated protein kinase
MMPs: Matrix metalloproteinases
MSU: Monosodium urate
MSU: Monosodium urate
NAFLD: Nonalcoholic fatty liver disease
NETs: Neutrophil extracellular traps
NF-κB: Nuclear factor kappa-B
NLR: NOD-like receptor
NLRP3: NLR family pyrin domain-containing 3
NOD: Nucleotide oligomerization domain
Nrf2: Nuclear factor erythroid 2 related factor 2
OPG: Osteoprotegerin
PPP1R3A: Protein phosphatase 1 regulatory subunit 3A
PPAR γ: Peroxisome proliferator-activated receptor γ
PTGS2: Prostaglandin endoperoxide synthase 2
RA: Rheumatoid arthritis
RANK: Receptor activator of NF-κB
RANKL: Receptor activator of nuclear factor-κB ligand
ROS: Reactive oxygen species
SCFAs: Short-chain fatty acids
Shi Zheng: Dampness syndrome
T2DM: Type 2 diabetes mellitus
TAK1: TGF-β-activated kinase 1
TC: Total cholesterol
TCM: Traditional Chinese medicine
TG: Triglyceride
TGs: Triglycerides
TLR4: Toll-like receptor 4
UA: Uric acid
URAT1: Urate transporter 1
VEGF: Vascular endothelial growth factor
VEGFR2: VEGF receptor 2
VLDL: Very-low-density lipoprotein
XOD: Xanthine oxidase
